# Statewide School-located Influenza Vaccination Program for Children 5–13 Years of Age, Hawaii, USA

**DOI:** 10.3201/eid1602.091375

**Published:** 2010-02

**Authors:** Paul V. Effler, Carl Chu, Howard He, Kate Gaynor, Steve Sakamoto, Marcia Nagao, Lisa Mendez, Sarah Y. Park

**Affiliations:** State of Hawaii Department of Health, Honolulu, Hawaii, USA; 1 Current affiliation: Department of Health, Perth, Western Australia, Australia.

**Keywords:** Influenza, school-located vaccinations, live attenuated influenza vaccine, trivalent influenza vaccine, pandemic response, viruses, Hawaii, research, expedited

## Abstract

New guidance recommends annual influenza vaccination for all children 5–18 years of age in the United States. During 2007–2008, Hawaii offered inactivated and live attenuated influenza vaccine at school-located clinics for grades kindergarten through 8. Most (90%) public and private schools participated, and 622 clinics were conducted at 340 schools. Of 132,775 children 5–13 years of age, 60,760 (46%) were vaccinated. The proportion vaccinated peaked at 54% for those 6 years of age and declined for older cohorts. More than 90% of schoolchildren transited the clinic in <10 minutes. A total of 16,920 staff-hours were expended; estimated cost per dose administered was $27 and included vaccine purchase and administration, health staffing resources, printing costs, data management, and promotion. This program demonstrates the feasibility of conducting mass school-located influenza vaccination programs in public and private schools statewide, as might be indicated to respond to pandemic influenza.

## Abstract 2

Nearly half of students in participating elementary and middle schools were vaccinated during 2007–2008.

Seasonal influenza reportedly results in 200,000 hospitalizations and 36,000 deaths annually in the United States (*1*). In addition, ≈31 million outpatient visits are attributable to influenza during seasonal epidemics; annual projected direct medical costs are $10.4 billion and lost earnings are $16.3 billion (*2*). Although schoolchildren are not considered at high risk of dying from influenza, annual illness attack rates in schoolchildren were >40% in some years (*3*,*4*). In addition, children 5–18 years of age may be the primary source of communitywide influenza transmission (*5**–**7*). Focused prevention of influenza infection among children may not only prevent childhood illness but also decrease school absenteeism and reduce the negative impact of influenza infection among working adults and elderly persons (*8*–*10*). Disease modeling suggests that if influenza vaccine was limited, as might be expected during a pandemic, vaccinating schoolchildren might be the most efficient approach to reducing overall numbers of influenza infections (*11*–*14*).

On February 27, 2008, the Advisory Committee on Immunization Practices (ACIP) expanded the recommended ages for annual influenza vaccination of children to include all children 6 months–18 years of age (*15*). Nationwide, this new recommendation added ≈30 million children to the cohorts targeted for annual influenza vaccination and invoked calls to consider alternatives to the physician’s office for administering the vaccine (*16**,**17*).

School-located influenza vaccination (SLIV) clinics have been proposed as a way to get more children vaccinated. Results from several trial SLIV programs indicate that vaccinated schoolchildren and their families experience lower rates of influenza-associated illnesses (*18**–**20*). However, many recent studies used only live attenuated influenza vaccine (LAIV), administered as a nasal spray, donated by the manufacturer (*6**,**21*). LAIV is currently not recommended for use in children with asthma or other underlying medical conditions that predispose them to complications from wild-type influenza infection; yet these potentially high-risk children may derive the most direct benefit from vaccination against influenza.

As a result, during the 2006–07 school year, the State of Hawaii Department of Health (DOH) conducted a pilot project to assess the feasibility of providing a choice of intranasal LAIV or intramuscular trivalent influenza vaccine (TIV) to students at 3 elementary schools. The pilot project achieved an overall vaccination rate of 35%. On the basis of this success, DOH conducted a statewide SLIV program during the 2007–08 influenza season. We present data on the logistics and outcomes of implementing this large-scale public health program; these data may be relevant to jurisdictions planning annual seasonal influenza vaccination programs for children and for responding to recent ACIP recommendations that place school-aged children among the top priority groups for receiving Influenza A (H1N1) 2009 Monovalent Vaccine (*22*,*23*).

## Methods

The 2007–08 SLIV program focused on all children 5–13 years of age in Hawaii. Public and private elementary and middle schools were identified through the databases of the Hawaii Department of Education, Hawaii Association of Independent Schools, and Hawaii Catholic Schools. All schools were invited to participate and could register online.

Influenza vaccination was voluntary and required written consent from the child’s parent or guardian. Information packets contained an explanatory letter, the 2007–08 Influenza Vaccine Information Statements for both vaccine formulations, and a consent form. A parent or guardian could specifically consent to their child receiving TIV or LAIV or indicate that either formulation was acceptable. To guide the parental decision, the consent form asked about potential contraindications to each formulation. If the child was <9 years of age and had not been vaccinated with 2 doses of influenza vaccine in any previous year (or if the parent, medical provider, or both were uncertain), parents were asked to consent to 2 doses of influenza vaccine administered >6 weeks apart. The packets were distributed to children at participating schools in August 2008; the consent forms were collected by the schools ≈4 weeks later. The packets were available online in 11 languages other than English commonly spoken in Hawaii (www.stopfluatschool.com). Families incurred no cost and received no incentive for participation. DOH provided the vaccine and all clinic supplies. School-based faculty and staff also were offered influenza vaccination at no cost through SLIV clinics.

Vaccines were administered at school during normal school hours over 59 working days from October 15, 2007, through January 31, 2008. School clinic dates and times were established through dialogue with the principal or other administrator of each school. School administrators at participating schools were asked to provide a large room for up to 4 hours (two 4-hour sessions >6 weeks apart for elementary schools), notify parents of the date and time of the scheduled vaccination clinic, and escort students to and from clinics. DOH staff, assisted by contract courier services, transported clinic supplies from DOH offices to each school 1 day before the clinic. DOH paid for all vaccine from state and federal funds and arranged all clinic staffing. No money was billed to third-party payers or collected on site.

Each school clinic required 1 clinic manager and >1 registration personnel and vaccinators, according to the number of vaccinations anticipated there. Staffing data (position, affiliation, hours spent) were determined prospectively and available for 92% of all clinics. For the remainder, we imputed their values from the mean value of the clinics with data available.

Participating DOH personnel, volunteers, and contract staff received DOH-developed training tailored to their respective program responsibilities. DOH verified that all vaccinators were licensed health professionals in good standing; the exception was nursing school students who worked under the on-site supervision of licensed faculty preceptors. In accordance with Hawaii state law, volunteers for DOH, including Medical Reserve Corps volunteers, were considered state employees for liability purposes.

We defined student age in years as date of first influenza vaccination administered by this program minus the child’s date of birth divided by 365.25. Student population size for the cohort of children aged 5–13 years residing in Hawaii on July 1, 2007, was obtained using publicly available census estimates (*24*).

To calculate school-level participation rates, the number of children enrolled in grades kindergarten through 8 (K–8) at individual schools was obtained from the Hawaii Council of Private Schools, Private School Enrollment Report 2007–2008, and the State of Hawaii Department of Education Official 2007–08 Enrollment. To calculate the proportion of school staff vaccinated, we obtained the total number of personnel employed at each school from the participating schools.

To calculate clinic throughput times, a nonrandomized subset of children were given a card documenting the time they arrived at the registration area. The card was collected and exit time noted as the child left the clinic area after vaccination. The difference between exit and arrival times, rounded to the nearest minute, was the clinic throughput time. Although we did not randomly select these students, we tried to obtain a representative sample by distributing the time-stamped cards to 10 children spread over the operational period at each of nearly 200 clinics, totaling ≈3% of vaccinated students.

We collected reports of potential adverse events after vaccination sent to the national passive surveillance system (Vaccine Adverse Event Reporting System) (*25*). We also maintained records of calls DOH received directly from parents, clinicians, or schools regarding potential adverse events.

Costs were estimated from the DOH perspective so that in-kind contributions from schools whose staff disseminated or collected consent materials and/or escorted students to clinics were not included. We estimated the cost of in-kind contributions of DOH staff and volunteers using 2007 salary data available from the US Department of Labor Bureau of Labor Statistics (*26*). Clinic manager hourly costs were defined as the national hourly wage for registered nurses at the 75th percentile ($35.18); vaccine administrator hourly costs were defined as the median hourly wage for registered nurses ($30.44), and registration personnel hourly costs were defined as the median hourly wage for medical records and health information technicians ($14.08).

We used Epi Info 2000 to calculate means and standard deviations for computing 95% confidence intervals (CIs); Epi Info version 6 χ^2^ test for trend was used to assess trends in proportions over successive age cohorts (*27*). Correlation coefficients were calculated by using Excel (Microsoft, Redmond, WA, USA).

## Results

Of 67,203 schoolchildren for whom consent was obtained, 63,153 (94%) received >1 doses of influenza vaccine. Of schoolchildren receiving at least 1 influenza vaccination, 1,078 (1.7%) were <5 years of age, 60,760 (96%) were 5–13 years of age, and 1,136 (1.8%) were >13 years of age; age information was missing for 179 (0.3%) children. Thus, 46% of 5–13-year-old children in Hawaii, as determined by census data (n = 132,775), were vaccinated against influenza at SLIV clinics.

Twenty-nine percent (n = 18,173) of children who received a first influenza vaccination at an SLIV clinic received a second dose, representing 60% of the 30,357 children <9 years of age who participated in the program. A total of 81,326 first or second vaccine doses were provided to children at SLIV clinics.

In addition, 9,306 (43%) of the 21,625 school staff members and 1,054 clinic volunteers were vaccinated at SLIV clinics, bringing the overall total number of influenza vaccinations administered through the program to 91,686. These 2 groups represented 14% of vaccinated persons.

A total of 340 (90%) of 377 elementary and middle schools in Hawaii participated in the program. This number constitutes 242 (96%) of 251 public schools and 98 (78%) of 126 private schools.

A total of 622 SLIV clinics were conducted; 345 (55%) were first-dose clinics during October through December 2007; 5 large schools had 2 first-dose clinics because of the number of vaccinees anticipated. Another 277 (45%) were second-dose clinics conducted at elementary schools in January 2008 to fully vaccinate children <9 years of age who had never received 2 doses of influenza vaccine in a prior year.

**Figure 1:**
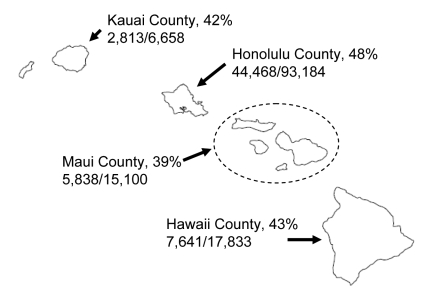
Number and proportion of children 5–13 years of age receiving >1 doses of influenza vaccine at school-located clinics, by county, Hawaii, USA, 2007–08 influenza season. Numerator is the number of children 5–13 years of age vaccinated in the program; denominator is the county population of children 5–13 years of age as of July 1, 2007.

Unless indicated otherwise, the following analyses are restricted to children 5–13 years of age. In Honolulu County, 48% of schoolchildren were vaccinated; in Hawaii’s other counties, 39%–42% were vaccinated (Figure 1).

**Figure 2:**
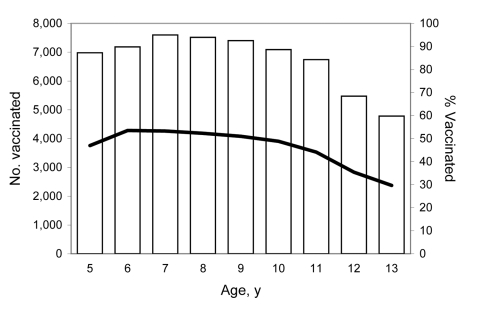
Number and percentage of children 5–13 years of age receiving at least 1 dose of influenza vaccine through a school-located clinic, by year of age, Hawaii, USA, 2007–08 influenza season. White bars indicate number of children vaccinated; black line indicates percentage of children vaccinated.

Vaccinations peaked at 54% for children 6 years of age. In successive age cohorts, the vaccination rates gradually declined to 30% for children 13 years of age (Figure 2).

**Figure 3:**
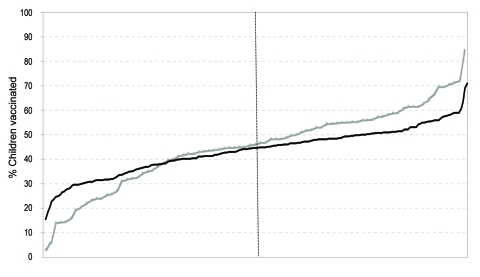
Vaccination rate ranking, by school (grades K–8), public and private schools, Hawaii, USA, 2007–08 influenza season. Black line indicates public schools ranked 1–208 (left to right); gray line indicates private schools ranked 1–83 (left to right). Vertical line indicates the median.

**Figure 4:**
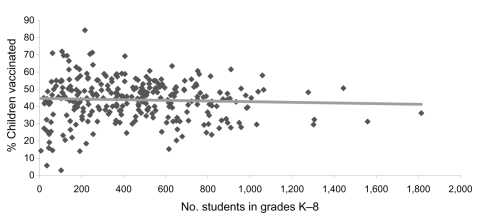
Proportion of children enrolled in grades K–8 at each school who received at least 1 dose of influenza vaccine, by school size, Hawaii, USA, 2007–08 influenza season. Linear fit trend (gray line) calculated by using Excel software (Microsoft, Redmond, WA, USA); r = –0.05.

Grade-level enrollment data required for calculation of school-level participation rates were available for 291 (86%) of the 340 participating schools. The proportion of children vaccinated at individual schools ranged from 3% to 84% (Figure 3). However, the mean proportion of schoolchildren vaccinated was similar between the 208 public (43.4%; 95% CI 42.1%–44.7%) and 83 private (45.0%; 95% CI 41.5%–48.5%) schools. School size, as measured by the number of students in grades K–8, correlated poorly with vaccination coverage rates obtained at the school (correlation coefficient: –0.05, Figure 4).

**Figure 5:**
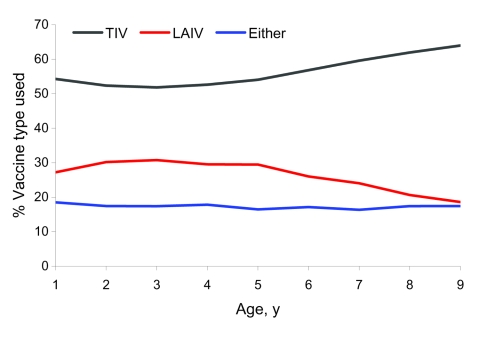
Proportion of children receiving at least 1 dose of influenza vaccine in school-located clinics, by age and vaccine formulation selected, Hawaii, USA, 2007–08 influenza season. LAIV, live attenuated influenza vaccine; TIV, trivalent Influenza vaccine; Either, parent or guardian consented to administration of either vaccine formulation to their child. N = 60,694; excludes 66 children for whom vaccine formulation data were not available.

Most (56%) parents selected TIV for their child; 27% chose LAIV; and 17% consented to have their child receive either formulation (p<0.001). Vaccine formulation preference did not differ significantly by student sex (p = 0.19). However, the trend for parents to select TIV (only) over LAIV or either vaccine in successive annual age cohorts was significant (p<0.001, Figure 5).

Clinic throughput times were obtained for 1,970 schoolchildren vaccinated at 199 separate clinics. Median throughput time was 4 minutes; >90% of schoolchildren transited the clinic in <10 minutes.

Physicians submitted vaccine adverse event reports for 3 children who received TIV through the school program; no events were medically serious or required hospitalization. In addition, DOH staff were informed of 4 other minor incidents, of which 3 were vasovagal syncopal episodes after TIV administration.

A total of 16,920 person-hours were expended to conduct 345 first-dose clinics and 277 second-dose clinics (Table 1). Mean duration of the first-dose clinic was 3.1 hours and typically required 1 clinic manager, 5 or 6 registration staff members, and 6 vaccinators. Second-dose clinics averaged 2.3 hours and used 2 registration staff members and 2 or 3 vaccinators**.**

**Table 1:** Person-hours expended to implement school-located influenza vaccination clinics, Hawaii, USA, 2007–08 influenza season*

Affiliation or time	First-dose clinics, n = 345				Second-dose clinics, n = 277				Total, n = 622		
	Man	Reg	Vac		Man	Reg	Vac		Man	Reg	Vac
PHN	827 (77)	–	1,633 (26)		520 (83)	–	541 (31)		1,347 (80)	–	2,174 (27)
DOH (non-PHN)	155 (15)	4,259 (72)	–		66 (10)	963 (75)	–		221 (13)	5,222 (73)	–
Contract nurses	86 (8)	59 (1)	2,831 (45)		37 (6)	57 (4)	994 (57)		123 (7)	116 (2)	3,825 (48)
Nursing school	–	73 (1)	1,172 (19)		3 (1)	14 (1)	31 (2)		3 (0)	87 (1)	1,203 (15)
Military	–	90 (2)	225 (4)		–	18 (1)	45 (3)			108 (2)	270 (3)
MRC	–	480 (8)	390 (6)		–	115 (9)	118 (7)			595 (8)	508 (6)
Other	–	934 (16)	63 (1)		–	8 (0)	114 (9)			1,048 (15)	70 (1)
Total hours	1,068	5,896	6,313		626	1,281	1,736		1,694	7,177	8,049
Mean hours/clinic	3.1	17.1	18.3		2.3	4.6	6.3		2.7	11.5	12.9

*Man, managers; Reg, registration; Vac, vaccinators; PHN, Public Health Nursing Branch staff, State of Hawaii Department of Health; DOH (non-PHN), other State of Hawaii Department of Health staff, including immunization program staff; nursing school, staff preceptors, and/or nursing school students; military, US Department of Defense healthcare personnel; MRC, Medical Reserve Corps staff from Oahu, Hawaii, Kauai, and Maui Medical Reserve Corps units; other, any other volunteer not affiliated with one of the above organizations. Values are no. (%) unless otherwise indicated.

DOH public health nurses accounted for 80% of clinic manager person-hours; other DOH staff provided almost 75% of person-hours for registration (Table 1). Contract nurses accounted for almost 50% of all vaccinator staff hours; DOH public health nurses and nursing school students filled most of the remaining need. Other organizations providing substantial staff support included the Hawaii Medical Reserve Corps and the US Department of Defense**.**

Program operation costs were estimated to be $2,480,493; nearly half was used to purchase vaccine. Forty-six percent of vaccine doses were acquired through the federal Vaccines for Children program (Table 2). The all-inclusive cost of administering 90,632 doses of influenza vaccine to participating children and school staff, comprising vaccine purchase and administration, healthcare staff resources, printing costs, data management, media promotion, and schoolchild participation rewards, was $27.37 per dose.

**Table 2:** Operation costs for statewide school-located influenza vaccination program, Hawaii, USA, 2007–08 influenza season*

Expenditure type	Cost, $	Percentage of total
Vaccine	1,198,403	48
Medical supplies (e.g., adhesive bandage, gloves, alcohol wipes, tissues)	135,000	5
Consent forms, VIS, other print materials	148,278	6
Data management (contracted data entry for consent forms)	42,230	2
Curriculum development and training materials	35,300	1
Public relations; television and radio public service announcements	216,706	9
Courier services to distribute forms and transport vaccine	19,300	1
Student rewards (stickers and hand stamps)	7,000	0
Staffing		
DOH staff hired to implement project	130,199	
Contract nursing services†	266,344	
Estimated cost of in-kind person-hour contributions, e.g., DOH, nursing schools, military, MRC	281,733	
Subtotal for staffing	678,276	*27*
Total	2,480,493	100

*VIS, vaccine information statement; DOH, State of Hawaii Department of Health; MRC, Medical Reserve Corps. †Contract nursing staff members were procured through an agreement with a private nurse staffing agency; the agency charged the department for a predetermined amount per hour per nurse provided.

## Discussion

This is the largest reported school-located influenza vaccination program in the United States and the only one to offer parents a choice of influenza vaccine formulation, i.e., TIV or LAIV (*28**–**31*). Through this effort, we vaccinated nearly half of all children 5–13 years of age in Hawaii against influenza.

Several valuable lessons can be learned. School vaccine coverage rates varied widely throughout the state, regardless of school size or school type (i.e., public vs. private), suggesting that other factors were important in determining acceptance of mass influenza vaccination at school. More work is needed to examine socioeconomic, cultural, and school-level characteristics that might influence participation in school-based vaccination programs.

A large proportion of public and private schools participated in the SLIV program. DOH promoted the program to school principals as a means to reduce influenza illness among students and staff, potentially resulting in decreased influenza-associated absenteeism. The high participation rates suggest that many principals believed the investment of student and staff time for the SLIV clinics was acceptable.

Vaccination rates declined among older, middle school–aged cohorts, suggesting that obtaining high influenza vaccination rates among high school-aged students may be challenging. This observation is consistent with low influenza vaccination rates attained at a high school in Hawaii during the 2006–07 school year and with our experience with school-located hepatitis B vaccination programs in Hawaii during the 1990s (Hawaii DOH, unpub. data).

The second-dose clinics were resource intensive. These clinics accounted for 45% of clinic sessions but for only 22% of vaccine administered. If the 2006 ACIP recommendation to vaccinate all children beginning at 6 months becomes widely implemented, successively fewer school-aged children would require a second dose of vaccine, potentially obviating the need for second-dose SLIV clinics.

When offered a choice, parents selected the injectable vaccine for their children over the nasally administered attenuated formulation by a 2-to-1 margin. Although some parents would have based their choices on differences in contraindications to the 2 vaccines, the magnitude of this difference and the increasing preference for TIV for older children suggests that many parents simply preferred the injectable formulation. We did not investigate the reasons but speculate that parents were more familiar with TIV. Anecdotal reports from clinics indicated that some children sought the injectable vaccine to demonstrate their machismo to their peers, and several children are known to have switched from the LAIV to the TIV waiting line, despite their parents’ wishes. Ongoing assessment of these attitudes and behaviors are important to determine whether the preference for injectable vaccine persists as parents become more familiar with nasally administered vaccine.

Despite substantial program promotion and favorable media attention, we did not reach our informal target of 50% coverage. Achieving higher rates may require changes in parental perceptions about the risk for influenza illness in children, with the potential benefits of vaccination outweighing any perceived potential risks to the child. Still, almost 30% of schools vaccinated more than half of their students in grades K–8 before the 2008 ACIP expanded recommendation (*1*). The new recommendation for school-aged children may facilitate achieving higher vaccination coverage in future years.

Each year, <30% of children 5–13 years of age are estimated to see a medical provider at any time during October through December, i.e., the usual time frame for administering influenza vaccine (*32*). Applying this figure to the population of children vaccinated in our SLIV program, we find that ≈42,532 additional visits to a physician’s office would be needed to reach the same level of coverage. Whether sufficient capacity exists to add these additional office visits to providers’ schedules can be debated; what seems clear, however, is that the SLIV clinics remove potentially substantial barriers to vaccination, i.e., the need for parents to secure transportation and take time away from work or other duties to bring their child into the provider’s office. SLIV clinics are convenient for parents and do not generate indirect costs through lost wages. Research among adults indicates that the mean cost of delivering influenza vaccinations is lower when they are provided in mass vaccination settings compared with scheduled office visits (*33*). More study is needed to comprehensively assess the costs and benefits of providing influenza vaccines to children at SLIV clinics.

Our study had at least 1 major potential limitation. Because the State of Hawaii has a relatively small population, one might question the generalizability of our findings to larger jurisdictions on the US mainland. However, with a population >900,000, Honolulu County has more residents than 98% of other US counties and one of the nation’s highest population densities (*34*). The SLIV program in Honolulu County generated a vaccination coverage rate comparable with that of the other 3 less populous counties, each with 60,000–172,000 residents. The consistency of our experience across large and small counties suggests that our results may be applicable to other jurisdictions considering implementation of an SLIV program.

This program demonstrates the feasibility of large-scale SLIV programs among children in elementary and middle schools offering both TIV and LAIV. No medically serious vaccine-associated adverse events were identified. Anecdotal information indicated that the program was well received; many parents and school staff requested that we repeat the SLIV clinics the following year. In response, the statewide program was continued for the 2008–09 school year and achieved similar rates of vaccination coverage for the 5–13-year-old cohort, as well as school participation rates, compared with the inaugural year (45% and 89%, respectively). The logistical effort required to conduct this program greatly improved our organizational capacity to conduct sustained mass vaccination clinics for children as might be required in response to a pandemic (*22**,**23*). In addition, this program laid the foundation for an ongoing evaluation of the potential effect of widespread influenza vaccination of school-aged children on the community at large.
